# Markerless Motion Capture for Human Movement Estimation Using Artificial Intelligence: A Systematic Review

**DOI:** 10.3390/pediatric18040083

**Published:** 2026-06-23

**Authors:** Georgina Domènech-Garcia, Xavier Marimon, Andoni Carrasco-Urribarren, Alejandro E. Portela, Caritat Bagur-Calafat

**Affiliations:** 1 Department of Physiotherapy, Universitat Internacional de Catalunya (UIC), 08195 Barcelona, Spain; acarrasco@uic.es; 2 Fundació Aspace Catalunya, 08038 Barcelona, Spain; 3 Department of Strength of Materials and Structural Engineering, Universitat Politècnica de Catalunya (UPC-12 Barcelona TECH), 08028 Barcelona, Spain; xavier.marimon@upc.edu; 4 Institute for Research and Innovation in Health (IRIS), 08028 Barcelona, Spain; 5 Bioengineering Institute of Technology, Universitat Internacional de Catalunya (UIC), 08195 Barcelona, Spain; aeportela@uic.es

**Keywords:** artificial intelligence, motion capture, data accuracy, markerless

## Abstract

**Background**: Artificial intelligence (AI)-driven markerless motion capture (MMC) technologies are increasingly being integrated into pediatric healthcare to improve the assessment and management of movement disorders. These video-based systems enable non-invasive motion analysis without wearable sensors, facilitating more natural movement assessment in children, particularly those with neurological or developmental conditions. **Objectives**: We evaluated the clinical applicability of AI-based MMC tools in pediatric settings for diagnosis, monitoring of motor development, and rehabilitation. **Methods**: This systematic review was registered in PROSPERO (CRD42024511787) and conducted by two independent reviewers, with a third reviewer resolving disagreements. The literature published between 2018 and 2025 was systematically searched. Studies involving pediatric populations or clinically relevant pediatric applications of MMC were included. **Results**: Of 1521 identified studies, 52 were finally selected. The included studies evaluated populations across a wide age range. However, seven of the included articles were specifically focused on underage populations. Infant studies primarily analyzed whole-body movements, emphasizing the relevance of global motor patterns in early development. OpenPose and AlphaPose were the most frequently used frameworks in pediatric research because of their automatic full-body key point detection, whereas DeepLabCut was commonly selected for its customizable labeling capabilities. Theia3D emerged as a promising clinically applicable solution with high accuracy. Most studies evaluated kinematic parameters as objective markers of motor performance and development. However, methodological heterogeneity and limited pediatric-specific validation remain important limitations. **Conclusions**: AI-driven MMC technologies show considerable potential to support objective, accessible, and child-friendly movement assessment in pediatric clinical practice.

## 1. Introduction

Artificial intelligence (AI) has become a hot topic of discussion in recent times. As technology continues to advance, AI has become an area of interest not only for experts but also for the public. However, there are still misconceptions about AI among clinicians. AI was conceived by McCarthy at the Dartmouth conference in 1956 as “the science and engineering of making intelligent machines” [1].

AI encompasses various subdisciplines, with machine learning being a prominent one [2]. It utilizes extensive datasets to discern interaction patterns among variables. Deep learning (DL), a subset of machine learning, emulates the neural operations of the human brain through multiple layers of artificial neuronal networks. This AI technique yields automated predictions from training datasets and finds compelling applications in image recognition, among other domains [3].

Among the diverse array of applications in everyday human activities, spanning financial services [4], automotive field [5,6], smart home technologies [6], and more, the incorporation of AI into healthcare stands out as holding tremendous potential. Research in this specific application predominantly focuses on addressing challenges related to cancer [7], nervous system disorders [8], and cardiovascular diseases [9], given their significant impact on disability and mortality rates. However, numerous articles have showcased promising advancements in utilizing AI for specific medical assessments [10,11,12,13,14,15] and training purposes [16].

Clinicians are looking for tools or methods to assess the progress of their patients easily and objectively. Nowadays, popular objective movement assessments systems are being developed in large laboratories equipped with extensive instrumentation [17]. Thanks to these AI techniques, low-cost alternatives are possible. Finding a robust and accurate way to measure human motion has always been the focus of researchers, and one of the great advantages of the use of DL-based methods is that they are extremely flexible and allow researchers to define what to track [18].

Motion estimation is a process used in computer vision and image processing to determine the motion of objects, features, or regions between consecutive frames in a sequence, such as a video or an image series. The integration of AI, particularly deep learning, has revolutionized motion estimation, offering promising advancements and future possibilities, like improved accuracy and robustness and end-to-end learning, as deep learning models can be trained end-to-end by directly learning to estimate motion from raw data without relying on handcrafted features [19].

For motion estimation, imaging is perhaps the most common and widely used because it allows non-invasive, high-resolution motion observations in a variety of settings [20,21]. Beyond estimating motion, AI can infer high-level semantics, like identifying specific actions, understanding a possible diagnostic, documenting disease progression, and evaluating treatment outcomes [22]. However, recording motion analysis in a special laboratory may not accurately reflect the actual movement patterns of individuals. These factors include the artificial nature of the setting, the influence of wearing specialized sensors or markers, and the potential for subjects to alter their behavior due to being observed, often referred to as the Hawthorne effect. Moreover, individuals may exhibit different behaviors in an unfamiliar laboratory or simulated environment during motion capture [23].

Recording motion analysis can be very expensive because it usually requires specialized laboratory equipment, like multiple high-speed cameras, reflective markers, and infrared (IR) cameras. These setups also need a controlled environment and trained professionals to operate and maintain them, which adds to the cost. A more affordable solution is to use an optic method that involves just a single video camera paired with a computer vision algorithm based on AI. This method can capture and analyze motion using standard video footage, without the need for expensive equipment or a controlled setting. Lam et al. 2023 [24] propose a solution to the issue above by advocating for the use of a smartphone camera in conjunction with an algorithm for analysis. They suggest that this combination could offer a feasible approach for evaluating patients’ daily movements through MMC, utilizing the capabilities of a smartphone and an advanced algorithm. 

Therefore, in this review, we emphasize exploring the optimal MMC technology as a crucial assessment tool in human motion analysis. Specifically, we focus on the AI software and algorithms employed in human biomechanical analysis and examine their key features. Additionally, we investigate the accuracy, clinical utility, and ease of use of these technologies in various contexts.

## 2. Materials and Methods

### 2.1. Search Strategy

This review was registered in the PROSPERO database (CRD42024511787). A comprehensive literature search was conducted across the following databases: Web of Science (WOS), PubMed/Medline, and IEEE Xplore. This systematic review was conducted in accordance with PRISMA guidelines (The PRISMA checklist is provided as Supplementary Materials (Table S2).

The equation search was the following: (“motion analysis” OR “movement analysis” OR “biomechanical assessment” OR “movement assessment” OR “motion tracking” OR “kinematic assessment” OR “motion capture” OR “motion estimation” OR “motion capture technology” OR “Computer Vision” OR “Video-based” OR “Pose Estimation”) AND (“artificial intelligence” OR “machine learning” OR “deep learning”) AND (markerless OR detectorless OR clusterless OR label-free). The final searching strategy was conducted up to 2 January 2026.

### 2.2. Inclusion and Exclusion Criteria

Studies were included if they met the following criteria: (1) presented any markerless motion capture AI algorithm for diagnosis or evaluation; (2) were published between 2018 and 2025; (3) were randomized or non-randomized clinical trials, validation studies, or observational studies, whether pilot or full-scale; and (4) were published in English or Spanish.

Studies were excluded if they met any of the following criteria: (a) involved non-human subjects; (b) were not markerless; (c) did not report accuracy or evaluation metrics; or (d) were of a different study type. Specifically, criterion (a) referred to studies involving animals or cadavers. Criterion (b) included studies that used external devices, relied on bounding-box detection rather than joint motion tracking, or employed multiple cameras or depth cameras. Criterion (d) referred to studies investigating a different topic or whose design did not correspond to the predefined eligible study types.

### 2.3. Selection Process

The studies collected by the searching strategy were screened by two independent authors (XMS, GDG). In cases of discrepancies, authors not involved in the initial screening acted as arbitrators.

**Figure 1:**
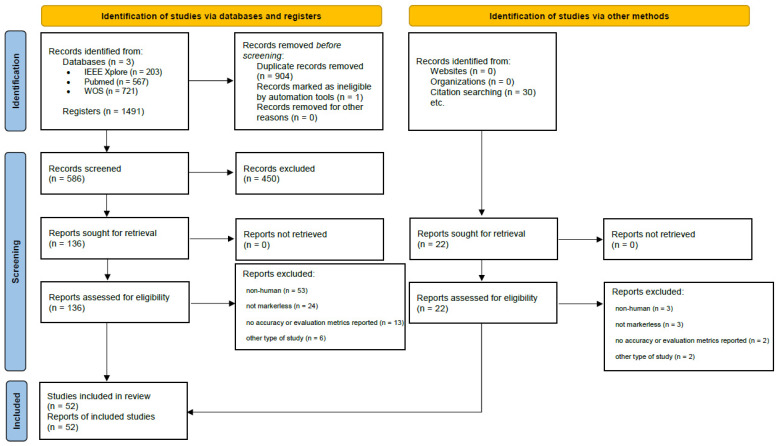
PRISMA 2020 flow diagram of the systematic review, which included searches of databases, registers, and other sources. We considered, if feasible to do so, reporting the number of records identified from each database or register searched (rather than the total number across all databases/registers) [25].

A total of 1521 articles were identified, with 1491 from databases search and 30 from citation searching. Duplicate records were removed using Mendeley Desktop v. 1.19.8 Reference Manager (Elsevier, Amsterdam, The Netherlands). According to their suitability, eligible articles were examined by title, abstract, and full text. The reasons for excluding studies were recorded. The selection process is summarized in the PRISMA flowchart in Figure 1.

### 2.4. Data Extraction and Analysis

Two reviewers were responsible for data extraction (XMS, GDG). A tabulated worksheet in Microsoft Excel (see Table S1 in Supplementary Materials) was used for the registration of relevant data from all studies included in this review. It includes the following research variables: (a) characteristics of the population; (b) characteristics of the video registration software and (c) AI motion tracking algorithm used, as well as (d) the accuracy results and (e) kinematic features; and (f) points of interest (PoI).

Of these, characteristics of the population refer to their age and health condition; characteristics of the video registration refer to its global dataset size, the AI motion tracking algorithm used, its training methodology, camera resolution, sampling frequency, the accuracy metrics reported, and the kinematic features, such as joint angles or velocity; and PoI refers to the analyzed body parts, such as upper or lower extremities, trunk, and whole body.

## 3. Results

A total of 1491 studies were found in the searches carried out in the WOS, PubMed/Medline, and IEE Xplore databases. In addition, 30 studies were found that could be included through other means, such as reviewing bibliographic references. A total of 904 duplicate titles and one other that could not be valued correctly were eliminated. Finally, after reading the title and abstract and applying the selection criteria of the present review, a total of 563 articles were eliminated, including 52 studies in Table S1 in Supplementary Materials.

Out of all the excluded articles, the majority focused on detecting laboratory characteristics like cancer diagnosis, which the authors deemed off topic. Among those that analyzed physical human motion tracking, various physical sensors were used, such as exoskeletons, robotic devices, or wearables, thus not meeting the criteria for being fully markerless. Others did not emphasize joint motion tracking but rather viewed participants as whole bodies (bounding box approach). Additionally, some studies employed multi-camera setups and/or depth cameras, which were not addressed in this article because they are not accessible in standard rehabilitation clinics using everyday consumer electronics. Moreover, many novel MMC systems are still in early development and reported mainly in conference proceedings, with limited validation within the scientific community.

### 3.1. Demographic Characteristics

The articles encompassed a broad age range, spanning from newborns to older individuals, with some studies focusing on specific age groups. Most of the studies (65%) included adult participants, whereas others included participants spanning multiple age groups, from children to adults [12,15,26,27,28]. Only a few studies were conducted exclusively with underage participants—infants [13,29,30,31,32] and children [33,34]. In the end, seven of these did not report the age range.

Nearly half of the articles (46%) examined motion tracking in healthy individuals. The remaining studies focused on populations with neurological conditions, including cerebral palsy [12,15,27,34,35], orthosis [36], Parkinson’s Disease [37,38,39], Multiple Sclerosis [40], preterm [13,30], human immunodeficiency virus encephalopathy [33], and post-stroke [41,42,43,44,45]. Other studies investigated populations with traumatic or musculoskeletal conditions, such as osteoarthritis [46,47]. In the end, 15% of the articles did not provide information about the health status under consideration.

### 3.2. Points of Interest

Considerable variability was observed in the regions of interest examined across studies. Most studies (41%) analyzed full-body kinematics, encompassing the entire body from the forehead to the great toe, and the lower extremities alone represented the second-most studied region (33%). Other studies focused specifically on the distal upper extremities [37,48,49] or both distal upper and lower extremities [38]. Some examined the trunk in combination with the upper extremities [27,50] or with the lower extremities [51,52,53,54,55]. In the end, 6% of the studies did not report their points of interest.

**Figure 2:**
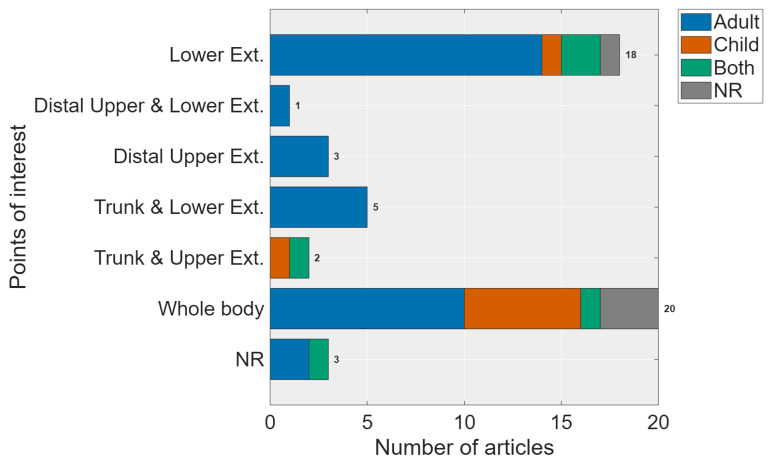
Number of articles describing different points of interest in each age group; Note: Ext.: extremity; NR: not reported.

The following graph in Figure 2 illustrates a trend where studies focusing on younger populations tend to analyze the entire body, due to their general movement objectives [13,29,30,31,32,33], whereas research involving older populations increasingly focuses on specific parts of the body or points of interest (trunk and upper or lower extremity for gait analysis, for example).

### 3.3. Recording Specifications

The range of camera resolutions employed in the studies varied, with the highest resolution set at 2704 × 1520 pixels [56] and the lowest at 520 × 520 pixels [39]. Most of the studies report 1920 × 1080 pixels while nearly half of them do not mention it. A diverse range of cameras was utilized in the research, including RGB cameras, smartphone cameras, and other types of cameras. Regarding the sampling frequency (Fs) from those that reported it, this was within a range of 25 Hz [15,26,27] up to 250 Hz [32], although 100 Hz was the most used (19%).

Although not all studies provided explicit details, there was variability in camera positioning, and it was adjusted according to the specific object/subject being recorded. While not all articles provided details on the dataset size of the collected videos, most authors separated them into training and validation datasets, with 60–90% of the collected data used for training and 10–20% of the remaining data used for validation. The smallest dataset size observed in the studies was six videos [57] and the highest 1140 videos [53].

### 3.4. Kinematic Feature Extraction

The kinematic features analyzed in the research varied according to the specific aims of the studies. Most studies (63%) focused on joint angle variations, followed closely by joint maker trajectories (50%). Other derived measures were also investigated, including joint velocity [12,27,39,58,59], joint acceleration [39], segment length [51], joint positions [32,51], and joint distances to a middle point of a reference line [26], which are features calculated mainly for shoulders, elbows, and wrists. One article analyzed the interlimb correlation [30].

The authors suggest the following features groups, extracted from all the articles features included in this review:

- Temporal regularity (T.R.): These time domain features measure timing and coordination aspects of movement, indicating how synchronized different body parts or movements are (mean interpeak intervals, interpeak variability, rhythm regularity).
- Joint marker trajectories (J.M.T.): These measures relate to a joint’s or body part’s spatial positioning and movement (joint position, distance to middle point, distance to line, trajectory deviation).
- Joint angles (J.A.): These measures pertain to the body’s segments of lengths and spatial relationships, which are directly related to joint angles (joint angle, segment length, mean amplitude, amplitude, variability, joint orientation).
- Interlimb correlation (I.C.): These measures refer to the relationship and coordination between the movements of different limbs (interlimb synchronization, interlimb complexity).
- Derivate measures (D.M.): These measures are more complex and derived from basic movement data, often reflecting higher-order characteristics of movement patterns (velocity, joint angular velocity, acceleration, curvature).

At the processing level, raw data frames are typically used. While most authors in this review work with raw data, some perform preprocessing through median filtering [51] and Gaussian filtering [30].

Statistical features of the data are also exploited to measure the interpeak variability of kinematic variables, such as mean amplitude, mean interpeak intervals, amplitude variability, and interpeak intervals variability [30].

Surprisingly, time and frequency domain features are not used in any of the publications examined in this systematic review. Only one publication used features based on the frequency domain [60]. A few articles were identified that used features based on non-linear measurements, chaos theory or information theory, and Sample Entropy measures [30]. Of interest are the features proposed by McCay et al. [60], where the kinematic parameters are encoded in 2D histograms, such as histograms of joint orientation and histograms of joint displacement. It is also interesting to note how Guayacan et al. proposed their results, where the curvature is encoded by a polar histogram scheme (dartboards) representing the magnitudes and directions of each feature [39].

### 3.5. Motion Estimation Algorithms

The predominant algorithm employed for motion estimation was the Convolutional Neural Network (CNN), utilized in 70% of the studies, where the type of network used was mainly ResNet [38] and FCNet [61] architecture. Several studies did not specify the deep learning algorithm employed [47,52,54,62,63,64,65]. Other approaches included Random Forest [59], Transformer [29,55], and ST-GCN [13]. Two studies did not provide information regarding the AI techniques used [46,66,67]. None of the articles reported the application of a dimensionality reduction technique to the data.

Regarding software tools, DeepLabCut (DLC) was the most used software (26%) followed by OpenPose (19%) and Theia3D (15%). Additional software solutions included MediaPipe [48,49,68,69,70], DensePose [39], AlphaPose [30,71], BlazePose [50], and KinaTrax [42,72]. Seven studies either customized the algorithm without specifying the tool or did not report this information. Moreover, Python is the primary programming language of such algorithm tools.

**Figure 3:**
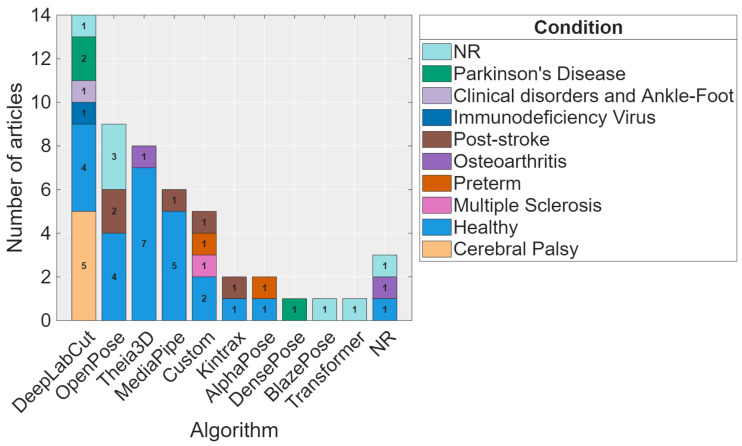
Algorithm distribution for each health condition. Note: number indicate the number of studies in which each algorithm was applied for each health condition.

Figure 3 presents which algorithm was used depending on health conditions being analyzed. It can be appreciated that DLC was the most used for its key points customization characteristics and OpenPose was the most used for its high range of accuracy. Moreover, Theia3D has gained prominence, providing end-to-end markerless motion capture pipelines that combine deep learning-based 2D key point detection with multi-view 3D reconstruction and biomechanical model fitting.

**Figure 4:**
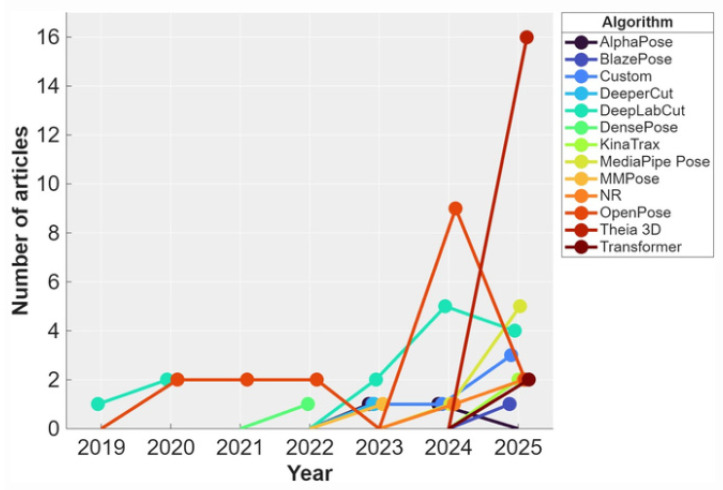
Evolution of algorithm usage.

Moreover, the temporal evolution in the use of each algorithm is presented in Figure 4. In 2024, there was an exponential increase in the number of newly introduced algorithm tools. Among them, Theia3D demonstrated the most substantial growth.

**Figure 5:**
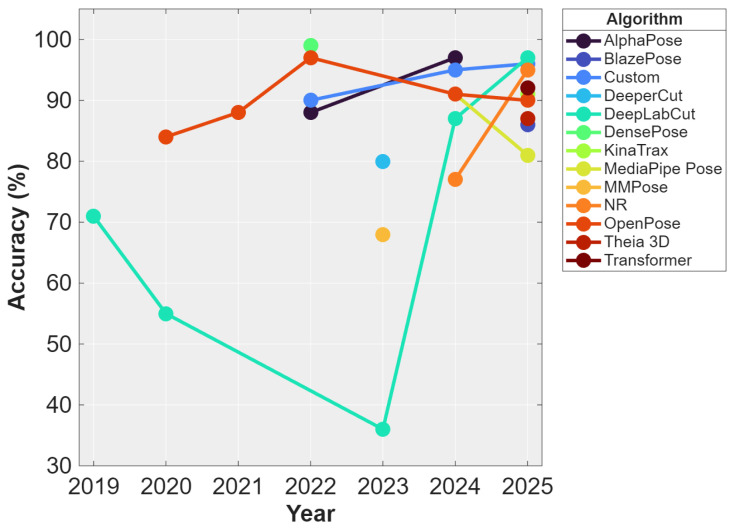
Evolution of algorithms accuracies. Note: Reported accuracy values should be interpreted with caution, as the underlying studies were not assessed for proper training methodology or healthy learning dynamics; thus, reported values may reflect overfitting or other methodological artifacts rather than true model generalizability.

This growth of algorithms usage is accompanied by their variability in accuracies, as represented in Figure 5. In the beginning, accuracies were reported in different ways: as accuracy percentages, r-squared (R2), Root Mean Squared Error (RMSE), Mean Absolute Error (MAE), and F-1 score. Accuracy was the most frequently used. Specifically, Shin HI. et al. reported their results with the r-square metric of 0.93 [30]. Another group of articles reported results in RMSE (2.93 pixels) [51] and others reported in MAE, ranging from 0.14 [26] up to 8.39 pixels [37].

### 3.6. Motion Estimation Accuracies and Their Training Method

Most reviewed studies did not undertake active model training, localized fine-tuning, or architectural optimization. Instead, they evaluated established pre-trained frameworks or commercial platforms operating in inference-only modes (e.g., based on COCO or MPII datasets), or they provided no information regarding training procedures. Only a small subset of studies implemented active machine learning processes involving model training or adaptation using the acquired data. [13,15,32,36,51,55,56,59,67,69].

Moreover, none of the articles provided evidence of learning curves while some of them selected transparent hyperparameters, either fully or partially [12,13,15,26,34,39,46,50,55,56,63,67,68,73,74,75,76].

However, when considering only studies employing appropriate training methodologies and reporting accuracies in a common metric (percentage), DensePose still achieves a higher percentage (99,62%), followed by AlphaPose (88–95%), Theia3D (70–95%), and OpenPose (84–88%). DLC had a major range of accuracy levels, from 93% up to 22.7%. Finally, DeeperCut was 80% accurate.

**Figure 6:**
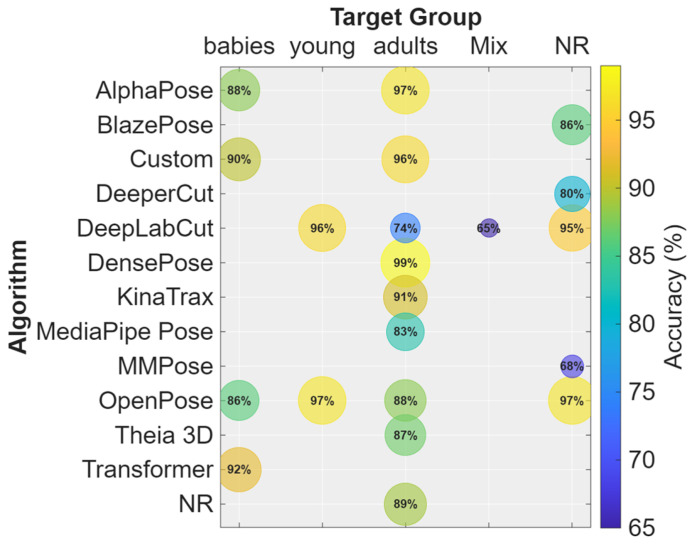
Accuracy distribution stratified by target age group. Note: Reported accuracy values should be interpreted with caution, as the underlying studies were not assessed for proper training methodology or healthy learning dynamics; thus, reported values may reflect overfitting or other methodological artifacts rather than true model generalizability.

These accuracies are held depending on the target age group, as demonstrated in Figure 6. Overall, most algorithm applications were predominantly evaluated in adult populations, where the highest concentration of studies and the widest range of reported accuracies were observed. In adults, several algorithms demonstrated high performance (DensePose, 99%) and achieved consistently strong results. Notably, DeepLabCut was one of the few tools consistently applied across multiple age categories (young, adults, and mixed samples), maintaining accuracies above 74% in all cases. In mixed-age samples, however, DLC showed a lower reported accuracy (65%).

In younger populations, OpenPose [32] and AlphaPose [30] (88%) achieved the highest reported accuracies in infant populations, whereas DeepLabCut was used in a children population, achieving a 96% of accuracy [33].

### 3.7. Validation

A significant portion of the articles outlined various validation methods. The k-fold-cross validation method was widely utilized [13,39], using mostly k = 5 folds of cross-validation. Additionally, studies commonly assessed inter-rater agreement between two raters and compared manual versus automatic labeling. [26,32]. Other approaches involved comparisons with an external device [38]; alternative motion tracking systems, such as Vicon [64]; other markerless algorithms [29]; the application of various traditional machine learning classifiers [31]; or evaluation against a CNN [37].

## 4. Discussion

Recent advances in AI have significantly expanded its application in various scientific fields, one of which is markerless pose estimation. This trend has been partly driven by the availability of open-source software. Despite these advancements, the use, comprehension, and interpretation of these technologies are not universally accessible. Therefore, this paper focuses on evaluating the performance and accuracy of various algorithm tools used in markerless pose estimation.

The present review compares several algorithm tools specifically developed for markerless human pose estimation, highlighting their distinctive features and suitability according to different research objectives. It includes articles from 2019 to 2025, as AI markerless technology is relatively new, with notable improvements in quality and quantity starting in 2024.

Across the included studies, most analyses focused on either the whole body or the lower extremities. In pediatric populations, assessments predominantly targeted whole-body motion, whereas in adults, evaluations covered a broader range of body segments (upper limb, lower limb, trunk, or full body). This distribution may reflect the clinical focus in children, where general movement patterns and global motor development are typically assessed. In such cases, predefined full-body markerless models may offer greater efficiency and practicality. Conversely, adult studies often investigate more specific biomechanical questions, requiring segmental or joint-specific analyses.

Regarding the populations studied, most participants were healthy individuals. Nevertheless, clinical populations were also represented, with cerebral palsy (CP) and post-stroke conditions being the most frequently investigated pathologies. This indicates a growing interest in validating markerless systems within neurorehabilitation and movement disorder contexts.

Considering the inclusion criteria—specifically studies employing purely markerless approaches without depth cameras—DeepLabCut (DLC) emerged as the most frequently used algorithm, followed by Theia3D. The versatility of DLC allows researchers to define and customize key points according to specific experimental needs, making it particularly suitable for tailored research applications. As one of the earliest and most widely adopted deep learning-based tools for markerless pose estimation, DLC has been extensively applied in biomechanics research over the past few years [77].

OpenPose supports full-body pose estimation, including facial landmarks, hand key points, and trunk key points, making it a versatile solution for comprehensive assessments [78]. Its continued use is largely attributed to its open-source framework and multi-person detection capabilities [79]. DensePose differs conceptually by providing dense 3D surface mapping rather than sparse anatomical key points, enabling more detailed surface-based analyses [39]. AlphaPose integrates symmetric heatmap aggregation and pose-guided proposals, achieving a favorable balance between precision and stability [80]. MediaPipe Pose, developed by Google, has demonstrated high sensitivity in clinical contexts and offers efficient real-time performance [81]. BlazePose and KinaTrax emerged more prominently in 2024 and are still under evaluation within the research community.

More recently, commercially available systems such as Theia3D have gained prominence, providing end-to-end markerless motion capture pipelines that combine deep learning-based 2D key point detection with multi-view 3D reconstruction and biomechanical model fitting [82].

When interpreting the reported accuracies of each algorithm, it is essential to consider the sample size and the characteristics of the dataset analyzed. Accuracy values may be influenced by the number of participants and the heterogeneity of the sample. In small cohorts, movement patterns may appear more homogeneous, potentially simplifying kinematic detection and increasing apparent accuracy. However, as sample size increases, inter-individual variability in anthropometrics, motor strategies, and movement quality also increases, which may introduce greater complexity and reduce performance consistency. In the reviewed studies, sample sizes ranged considerably from as few as five participants [48] to as many as 1176 individuals [55], which should be considered when comparing reported outcomes.

DeepLabCut (DLC) exhibited the widest variability in reported accuracies, particularly in studies including mixed-age populations, with values ranging from 17% to 99%. This broad range suggests potential sensitivity to heterogeneous datasets and diverse movement characteristics. Notably, DLC was the only algorithm consistently applied across such a wide age spectrum, reflecting researchers’ confidence in its adaptability and customizable framework [12,15,26,27,28].

OpenPose maintained relatively stable and generally high accuracy values over time, ranging from 68% to 99% [45,57] and demonstrating robustness across different study designs and populations. Overall, most algorithm tools reported accuracy values above 75%, indicating that markerless pose estimation has reached a level of performance that is broadly acceptable for many biomechanical and clinical research applications, although variability persists depending on population characteristics and methodological factors.

The authors recommend comparing the evaluations of two raters and assessing the differences between manual and automatic labeling. This recommendation is based on the opinion of the clinician writing, emphasizing the ease of accessibility for clinical use and the parallels observed in other types of studies. Moreover, some algorithm tools still lack sufficient clinical and comprehensive validation; therefore, further studies are needed to establish clear criteria for their optimal use.

Comprehensive details regarding the number of full-length videos utilized, their durations, and the allocation between training and validation sets were often lacking in the reviewed studies. The predominance of datasets used for training underscores the necessity for more consistent reporting practices in research. Furthermore, the absence of a standardized accuracy reporting format has led to a wide range of evaluation metrics being employed, complicating direct comparisons across studies. However, all algorithms reviewed demonstrate excellent accuracy, with accuracy percentages > 80%, R2 values > 90%, and F1-scores > 0.9, indicating high effectiveness. Given the comprehensive review of the literature, if the authors were tasked with recommending an evaluation metric for markerless motion estimation using AI for clinicians, the suggestion would be to adopt percentage accuracy as the gold standard measure. This recommendation is based on the widespread use of percentage accuracy in various studies, indicating its widespread acceptance and applicability in the field. Moreover, the familiarity and simplicity of percentage accuracy make it easily interpretable for both researchers and clinical practitioners, enhancing the accessibility and utility of study.

The most used features are time domain measurements, i.e., joint-related kinematic features. Frequency domain or time-frequency measurements are absent. Non-linear measurements are very few and are based on entropy. In this review, it becomes evident that AI-based models, particularly CNNs, are highly effective at learning complex patterns in motion data. CNNs excel at processing and interpreting spatial information, making them adept at handling visual inputs and in recognizing intricate details despite variations in lighting or partial occlusions. These CNN models are less affected by noise and environmental changes compared to traditional methods. Their ability to learn and adapt from diverse and noisy data leads to more precise motion estimation. Consequently, the use of CNNs in motion analysis results in improved accuracy and robustness, enabling more reliable and consistent performance in various real and clinical conditions.

In the field of motion analysis, AI offers capabilities that go beyond simple motion estimation by enabling the extraction of high-level semantic information. This advanced functionality allows AI systems to not only track and quantify motion but also recognize and categorize specific actions and behaviors, which is particularly valuable for diagnostics, identifying abnormalities in motion patterns that may indicate the presence of certain health conditions. Overall, AI’s ability to interpret movement data at a high level of abstraction enhances its utility in clinical practice, offering deeper insights that support accurate diagnosis, effective monitoring of disease progression, and optimal evaluation of treatment efficacy.

Particularly in pediatric populations, AlphaPose, OpenPose, and DeepLabCut are among the most widely used pose estimation frameworks due to their distinct methodological characteristics. AlphaPose and OpenPose provide predefined full-body key point models, whereas DeepLabCut offers a flexible, customizable framework for tracking user-defined anatomical landmarks. In babies, research typically focuses on full-body analysis, whereas in children and adolescents, studies more often concentrate on specific body segments or functional tasks, such as gait or grasping. Therefore, the selection of these tools generally depends on the study objectives, particularly on whether the focus is on whole-body motor coordination or on the movement of specific body segments.

Deep learning models offer a significant advantage in motion estimation by enabling end-to-end training directly from raw data. This means that these models learn to estimate motion without the need for manually designed features or preprocessing steps. Instead, the models automatically extract relevant features and patterns from the raw input data, such as images or video frames.

End-to-end learning facilitates real-time or live-motion estimation by processing raw data directly through a unified model, which enables us to rapidly interpret and analyze data, delivering motion estimates with minimal delay. The model’s ability to efficiently handle diverse conditions and variations ensures accurate performance even in dynamic environments. By integrating all processing tasks into a single model, it reduces latency and provides timely results, making it ideal for live diagnostics.

Unfortunately, the use of AI among clinicians is not yet widely standardized due to its complexity and challenges in understandability. This complexity makes it difficult for healthcare professionals to integrate AI into their workflows effectively. The challenge in the future lies in making AI outputs clear and actionable for clinicians who may not have a deep technical background. As a result, the widespread use of AI in clinical settings remains uneven, with many healthcare providers struggling to fully leverage its capabilities.

Moreover, to effectively integrate AI into clinical practice, the authors recommend making clear which PoI is intended to analyze and the age and health condition of this population before selecting an algorithm for motion analysis. It is important that AI systems present results in a way that clinicians can easily understand. This means designing AI outputs to be straightforward and actionable, so that healthcare professionals can quickly interpret and use the information in their decision-making processes. In addition, having medical engineers or data scientists as part of the clinical team can address more complex aspects of data analysis. These professionals code and train complicated AI models and bridge the gap between advanced AI algorithms and practical clinical applications. Their expertise ensures that AI findings are translated into actionable medical insights, increasing the overall utility of AI in healthcare.

Lam W et al. (2023) conducted a comparable review using different inclusion criteria (e.g., depth-sensing Kinect cameras) [24]. They concluded that MMC technology holds promise both as an assessment tool and symptom detection, potentially supporting AI-based early disease screening. Our review builds on this work, confirming their conclusions and providing deeper, software-specific analysis [24].

A major limitation identified across the reviewed literature is the widespread lack of transparent reporting regarding the underlying learning dynamics of the deployed machine learning algorithms. In studies focused on model training or customization, final accuracy metrics cannot be considered inherently trustworthy indicators of generalizability without explicit evidence of proper training methodologies, such as data separation protocols, evaluation of learning curves, and robust hyperparameter selection. High accuracy outcomes can frequently mask methodological errors, including data leakage between training and testing sets or severe overfitting to specific laboratory settings. Furthermore, for those studies that evaluate pre-trained off-the-shelf systems, the reported validity metrics remain deeply dependent on the demographic and contextual characteristics of the original training repositories (e.g., COCO or MPII datasets). Consequently, readers and clinicians must interpret aggregate performance metrics with appropriate caution, as the available literature rarely provides a standardized auditing of a model’s internal learning dynamics or resistance to overfitting artifacts.

Overall, the field is evolving rapidly, with a clear shift toward more automated, computationally efficient, and clinically adaptable deep learning-based markerless motion capture systems. High quality systematic reviews of this topic should be conducted periodically and include a broader range of databases to ensure the literature remains up to date and its clinical application.

## 5. Conclusions

Among the algorithms analyzed, DeepLabCut was the most frequently utilized algorithm across the reviewed studies in both pediatric and adult populations. OpenPose often demonstrated higher and more stable accuracy values and was more frequently applied in pediatric populations. Moreover, Theia3D is also emerging as a prominent commercial solution, benefiting from its reported high accuracy and integrated end-to-end commercial workflow. However, comparisons of reported algorithm performance across studies should be interpreted with caution because of methodological heterogeneity, differences in datasets, outcome measures, and evaluation procedures, as well as the limitations discussed throughout the manuscript. These factors may influence reported accuracies and hinder direct comparisons between studies. Furthermore, the present review did not assess whether the included studies employed appropriate training methodologies or healthy learning dynamics, as this was beyond the scope of the analysis.

In line with these limitations, the comparison of accuracy metrics across studies remains challenging due to the heterogeneity of outcome measures. Different units (e.g., pixel error, millimeters, mean per-joint position error, percentage of correct key points, correlation coefficients) limit direct comparability between systems. Therefore, there is a clear need for methodological standardization. Based on the findings of this review, adopting percentage accuracy as a common reporting metric for AI-based markerless motion estimation may improve interpretability and facilitate comparisons across studies. Percentage-based metrics are widely understood, relatively intuitive, and potentially more accessible for both researchers and clinical practitioners.

In conclusion, AI-driven markerless pose estimation demonstrates strong potential and is evolving at a rapid pace. Nevertheless, a significant gap remains between technological development and clinical implementation and validation. Many clinicians may lack the technical background required to critically interpret AI-derived kinematic outputs. Integrating medical engineers or data scientists into clinical and research teams could facilitate data interpretation, improve methodological rigor, and accelerate translation into practice. Continued interdisciplinary collaboration will be essential to ensure that advances in markerless motion capture meaningfully benefit research and clinical care.

## Data Availability

No new data were created.

## Supplementary Materials

The following supporting information can be downloaded at: https://www.mdpi.com/article/10.3390/pediatric18040083/s1, Table S1: Extraction table [10,12,13,15,20,26,27,28,29,32,33,34,36,37,38,39,40,41,42,43,44,45,47,48,49,50,51,52,53,54,55,56,57,59,60,62,64,65,67,68,69,70,71,72,73,74,75,80,83,84,85,86]; Table S2: PRISMA checklist.

## Abbreviations

The following abbreviations are used in this manuscript:

AI	Artificial intelligence
MMC	Markerless motion capture
DL	Deep learning
Ext	Extremity
NR	Not reported
TR	Temporal regularity
JMT	Joint marker trajectory
JA	Joint angle
DM	Derivative measure
CNN	Convolutional neural network
R2	Root square
RMSE	Root mean squared error
MAE	Mean absolute error
DLC	DeepLabCut
COCO	Common objects in context
MPII	Max Planck Institute for Informatics
