# Supplementary material for: Markerless Motion Capture for Human Movement Estimation Using Artificial Intelligence: A Systematic Review
Source: Pediatr Rep. 2026 Jun 23;18(4):83. doi: 10.3390/pediatric18040083 (PMC13398251; doi:10.3390/pediatric18040083)
Supplement: Supplementary file 1 [file pediatrrep-18-00083-s001.zip › pediatrrep-4337605-supplementary.pdf]

Supplementary Materials

Table S1. Extraction table.

Author, Year	Title	Year	n	Age Range	Health Condition	Global Dataset Size	Software	Algorithm	Accuracy (%)	Kinematic Features	Points of Interest
Alammari B. et al., 2025 [42]	Validity of AI-Driven Markerless Motion Capture for Spatiotemporal Gait Analysis in Stroke Survivors	2025	19	Adults	Post-stroke	105 videos	KinaTrax	CNN	90.0–95.0	Temporal regularity	Lower extremity
Asaeda M. et al., 2024 [70]	Reliability and validity of knee valgus angle calculation at single-leg drop landing by posture estimation using machine learning	2025	15	Adults	Healthy	150 videos	MediaPipe Pose	CNN	44.0	Joint angles<br>Derivative measures	Lower extremity
Boldo M. et al., 2024 [73]	On the reliability of single-camera markerless systems for overground gait monitoring	2024	8	Adults	Healthy	160 videos	OpenPose	CNN	88.0	Joint marker trajectories<br>Derivative measures	Whole body
Cameron-Whytock H. et al., 2025 [54]	Marker based and markerless motion capture for equestrian rider kinematic analysis: A comparative study	2025	10	Adults	Healthy	800 videos	Theia3D	DL-based	94.0	Joint angles	Trunk and lower extremity
Carriere J. et al., 2025 [56]	Can Machine Learning Enhance Computer Vision-Predicted Wrist Kinematics Determined from a Low-Cost Motion Capture System?	2024	NR	NR	Healthy	NR	DeepLabCut	CNN	90.0	Joint angles	Whole body
Ceriola L. et al., 2024 [71]	Comparative Analysis of Markerless Motion Capture Systems for Measuring Human Kinematics	2024	10	Adults	Healthy	120 videos	AlphaPose	CNN	95.0–99.0	Joint angles	Lower extremity
Cho S. et al., 2025 [67]	Video-estimated peak jump power using deep learning is associated with sarcopenia and low physical performance in adults	2025	223	Adults	NR	673 videos	NR	NR	95.0	Temporal regularity	Whole body
Cronin N.J. Et al., 2024 [83]	Feasibility of OpenPose markerless motion analysis in a real athletics competition	2024	12	NR	NR	12 videos	OpenPose	CNN	95.0	Joint angles<br>Derivative measures	Whole body

Cronin N.J. et al., 2019 [51]	Markerless 2D kinematic analysis of underwater running: A deep learning approach	2019	21	Adults	NR	530 images	DeepLabCut	CNN	70.8	Joint angles<br>Joint marker trajectories	Trunk and lower extremity
Cunha B. et al., 2025 [50]	Smartphone-Based Markerless Motion Capture for Accessible Rehabilitation: A Computer Vision Study	2025	15	NR	NR	138 videos	BlazePose	DTW	86.0	Joint angles<br>Joint marker trajectories	Trunk and upper extremity
Dinh H. et al., 2025 [48]	Proof of Concept and Validation of Single-Camera AI-Assisted Live Thumb Motion Capture,	2025	5	Adults	Healthy	30 videos	MediaPipe Pose	CNN	98.8	Joint angles<br>Joint marker trajectories	Distal upper extremity
Eken M.M. Et al., 2025 [33]	Markerless Upper Body Movement Tracking During Gait in Children with HIV Encephalopathy. A pilot study	2025	10	Child	HIVE	59 videos	DeepLabCut	CNN	96.0	Joint angles<br>Joint marker trajectories	Whole body
Falisse A. et al., 2025 [55]	Marker Data Enhancement for Markerless Motion Capture	2025	1176	Adults	Healthy	160 videos	Custom	Transformer	95.2–97.7	Joint angles<br>Derivative measures	Trunk and lower extremity
Galasso S. et al., 2024 [74]	A Novel Measurement Procedure for Error Correction in Single Camera Gait Analysis	2024	29	Adults	Healthy	29 videos	OpenPose	CNN	93.0	Joint angles<br>Derivative measures	Lower extremity
Gama F. et al., 2025 [29]	Automatic infant 2D pose estimation from videos: comparing seven deep neural network methods	2025	NR	Child (babies)	NR	8 videos	Transformer	Transformer	91.8	Joint marker trajectories	Whole body
Groos D. et al., 2022 [13]	Development and Validation of a Deep Learning Method to Predict Cerebral Palsy From Spontaneous Movements in Infants at High Risk	2022	557	Child (babies)	Preterm	557 videos	Custom	ST-GCN	90.0	Joint angles<br>Joint marker trajectories<br>Derivative measures	Whole body
Guayacan L.C. et al., 2022 [39]	Quantification of Parkinsonian Kinematic Patterns in Body-Segment Regions During Locomotion	2022	22	Adults	PD	176 videos	DensePose	CNN	99.0	Derivative measures	NR
Haberfehlner H. et al., 2024 [15]	A Novel Video-Based Methodology for Automated Classification of Dystonia and Choreoathetosis in Dyskinetic Cerebral Palsy During a Lower Extremity Task	2024	43	Both	CP	86 videos	DeepLabCut	CNN	60.0	Joint angles<br>Joint marker trajectories	Lower extremity
Haberfehlner H. et al., 2023. [26]	Towards automated video-based assessment of dystonia in dyskinetic cerebral palsy: A novel approach using markerless motion tracking and machine learning	2023	34	Both	CP	187 video sequences	DeepLabCut	CNN	55.0	Joint angles<br>Joint marker trajectories	Whole body

Haddas R. et al., 2025 [47]	Comparison of Markerless and Conventional Marker-Based Shoulder Kinematics Models During Activities of Daily Living in Patients With Glenohumeral Osteoarthritis	2025	100	Adults	Osteoarthritis	2118 videos	Theia3D	DL-based	81.0–99.0	Joint angles<br>Derivative measures	Whole body
Homlong E. et al., 2025 [34]	Addressing Occlusions and Pose Challenges in Clinical Gait Analysis: A Robust 3D-to-2D Motion Pipeline	2025	238	Child	CP	2284 videos	DeepLabCut	CNN	95.0	Joint marker trajectories<br>Joint angles	Lower extremity
Hu B. et al., 2025 [41]	Dual-Camera Markerless Motion Capture System for Precise Lower-Limb Kinematic Analysis in Osteoarthritis	2025	152	Adults	Osteoarthritis	152 videos	NR	NR	95.0	Joint angles<br>Temporal regularity	Lower extremity
Huijsmans H. et al., 2023 [12]	Markerless motion tracking to assess upper limb dyskinesia in children and young adults with cerebral palsy	2023	51	Both	PD	35 videos	DeepLabCut	CNN	17.0	Joint angles<br>Joint marker trajectories	NR
Jatesiktat P. et al., 2025 [75]	Anatomical-Marker-Driven 3D Markerless Human Motion Capture	2025	200	Adults	Healthy	5.39 million images	Custom	R-CNN, GAN	98.7	Joint marker trajectories	Whole body
Kearney J. et al., 2025 [65]	Comparison of Theia3D and the conventional gait model in typically developing children and adults in a clinical gait laboratory	2025	34	Adults	Healthy	NR	Theia3D	DL-based	98.0–50.0	Joint angles<br>Derivative measures	Whole body
Kim T. et al., 2025 [68]	Suggestion for Camera Location in Monocular Markerless 3D Motion Capture System: Focused on Accuracy Comparison With Marker-Based System for Upper Limb Joints	2025	10	Adults	Healthy	NR	MediaPipe Pose	CNN	93.0	Joint angles<br>Joint marker trajectories	Whole body
Li Z. et al., 2025 [80]	Impact of Multi-View Fusion and Biomechanical Modeling on Markerless Motion Tracking, IEEE transactions on bio-medical engineering	2025	23	Adults	Healthy	115 videos	Theia3D	DL-based	94.3–95.6	Joint angles<br>Joint marker trajectories	Lower extremity
Liu L. et al., 2024 [57]	Real-time pose estimation and motion tracking for motion performance using deep learning models	2024	NR	NR	NR	6 videos	OpenPose	CNN	97.3–99.3	Joint markers trajectories	Whole body
McCay K.D. et al., 2020 [60]	Abnormal Infant Movements Classification With Deep Learning on Pose-Based Features	2020	NR	Child (babies)	NR	12 videos	OpenPose	CNN	84.0	Joint angles	Whole body

Milone D. et al., 2024 [84]	MocapMe: DeepLabCut-Enhanced Neural Network for Enhanced Markerless Stability in Sit-to-Stand Motion Capture	2024	20	Adults	Healthy	NR	DeepLabCut	CNN	90.0–95.0	Joint marker trajectories<br>Derivative measures<br>Temporal regularity	Lower extremity
Moro M. et al., 2025 [40]	Markerless Video-Based Gait Analysis in People With Multiple Sclerosis	2024	50	Adults	MS	18 videos	Custom	CNN	95.0	Joint angles	Lower extremity
Mundt M. et al., 2024 [85]	Automating Video-Based Two-Dimensional Motion Analysis in Sport? Implications for Gait Event Detection, Pose Estimation, and Performance Parameter Analysis	2024	15	Adults	Healthy	298 videos	OpenPose	CNN	70.0–93.0	Joint marker trajectories	Whole body
Nishikawa N. et al., 2025 [52]	Comparison of kinematics between markerless and marker-based motion capture systems for change of direction maneuvers	2025	23	Adults	Healthy	115 videos	Theia3D	DL-based	75.0–94.0	Joint angles	Trunk and lower extremity
Panconi G. et al., 2025 [86]	DeepLabCut custom-trained model and the refinement function for gait analysis	2025	38	Adults	Healthy	40 videos	DeepLabCut	CNN	99.0	Temporal regularity	Whole body
Park J. et al., 2024 [10]	Markerless Kinematic Data in the Frontal Plane Contributions to Movement Quality in the Single-Leg Squat Test: A Comparison and Decision Tree Approach	2024	91	Adults	Healthy	273 videos	NR	NR	76.9	NR	NR
Peng Y. et al., 2025 [44]	Analysis of Pelvis and Lower Limb Coordination in Stroke Patients Using Smartphone-Based Motion Capture	2025	29	Adults	Post-stroke	370 videos	OpenPose	CNN	89.5	Joint marker trajectories<br>Temporal regularity	Lower extremity
Reich S. et al., 2021 [32]	Novel AI driven approach to classify infant motor functions	2021	51	Child (babies)	Healthy	1784 videos	OpenPose	CNN	88.0	Joint marker trajectories	Whole body
Schoenwether B. et al., 2025 [72]	Reliability of artificial intelligence-driven markerless motion capture in gait analyses of healthy adults	2025	9	Adults	Healthy	135 videos	KinaTrax	CNN	89.6	Joint marker trajectories<br>Temporal regularity	Lower extremity
Shin H. et al., 2022 [20]	Deep learning-based quantitative analyses of spontaneous movements and their association with early neurological development in preterm infants	2022	65	Child (babies)	Preterm	NR	AlphaPose	CNN	88.0	Joint angles<br>Derivative measures<br>Interlimb correlation	Whole body
Shin J.H. et al., 2020 [38]	Objective measurement of limb bradykinesia using a marker-less tracking algorithm with 2D-video in PD patients	2020	29	Adults	PD	106 videos	DeepLabCut	CNN	93.0	Joint angles<br>Temporal regularity	Distal upper extremity and

											distal lower extremity
Tahara A. et al., 2025 [69]	Predicting Walkway Spatiotemporal Parameters Using a Markerless, Pixel-Based Machine Learning Approach	2025	17	Adults	Healthy	170 videos	MediaPipe Pose	CNN	89.5	Joint marker trajectories<br>Temporal regularity	Whole body
Thomas C. et al., 2025 [62]	Comparison of Marker-Based and Markerless Motion Capture Systems for Measuring Throwing Kinematics	2025	13	Adults	Healthy	65 videos	Theia3D	DL-based	70.0–90.0	Joint angles<br>Joint marker trajectories<br>Temporal regularity	Lower extremity
Van der Waard et al., 2024 [36]	Motion tracking with automated pose estimator can enhance ankle-foot-orthoses alignment	2024	30	NR	Neurological disorders and Ankle-Foot Orthoses	30 videos	DeepLabCut	CNN	99.0	Joint angles<br>Joint marker trajectories	Lower extremity
Vanmechelen I. et al., 2024 [27]	Markerless motion analysis to assess reaching-sideways in individuals with dyskinetic cerebral palsy: A validity study	2024	51	Both	CP	408 videos	DeepLabCut	CNN	89.0–99.0	Joint angles<br>Derivative measures<br>Temporal regularity	Trunk and upper extremity
Verhoeven M. et al., 2025 [28]	From marker to markerless: Validating DeepLabCut for 2D sagittal plane gait analysis in adults and newly walking toddlers	2025	28	Both	Healthy	56 videos	DeepLabCut	CNN	97.0	Joint angles<br>Derivative measures	Lower extremity
Wagh V. et al., 2024 [49]	Quantifying Similarities Between MediaPipe and a Known Standard to Address Issues in Tracking 2D Upper Limb Trajectories: Proof of Concept Study	2024	10	Adults	Healthy	115 videos	MediaPipe Pose	CNN	89.0–93.8	Joint marker trajectories	Distal upper extremity
Wagh V. et al., 2025 [43]	Using MediaPipe to track upper-limb reaching movements after stroke: a proof-of-principle study	2025	7	Adults	Post-stroke	175 videos	MediaPipe Pose	CNN	75.0–85.0	Joint marker trajectories<br>Temporal regularity	Whole body
Williams S. et al., 2020 [37]	The discerning eye of computer vision: Can it measure Parkinson's finger tap bradykinesia?	2020	39	Adults	PD	137 videos	DeepLabCut	CNN	16.1	Joint marker trajectories<br>Derivative measures<br>Temporal regularity	Distal upper extremity
Yang C. et al., 2025 [64]	Comparison of lower limb kinematic and kinetic estimation during athlete jumping between markerless and marker-based motion capture systems	2025	14	Adults	Healthy	126 videos	Theia3D	DL-based	94.6	Joint angles	Lower extremity

Yang Z. et al. 2025 [45]	A graphical user interface for editing keypoints from human pose estimation algorithms	2024	23	Adults	Post-stroke	23 videos	OpenPose	CNN	68.0	Joint marker trajectories<br>Joint angles	Lower extremity
Yoma M. et al., 2025 [53]	Between-Day Reliability of Kinematic Variables Using Markerless Motion Capture for Single-Leg Squat and Single-Leg Landing Tasks	2025	19	Adults	Healthy	1140 videos	Theia3D	CNN	89.5	Joint angles	Trunk and lower extremity
Zhang L. et al., 2025 [59]	Towards Clinical Application of Enhanced Timed Up and Go with Markerless Motion Capture and Machine Learning for Balance and Gait Assessment	2025	70	Adults	Post-stroke	210 videos	Custom	Random Forest	94.0	Joint marker trajectories<br>Joint angles<br>Derivative measures<br>Temporal regularity	Lower extremity

Note: Age range: Child (babies), >1 year old; Child, 8–11 years old; Child, (adolescents) 11–13 years old; Adults >18 years old; Both, mixed ages (children and adults). Health condition: HIVE, human immunodeficiency virus encephalopathy; PD, Parkinson's disease; CP, cerebral palsy; MS, multiple sclerosis. Algorithm: DL, deep learning; CNN, convolutional neural network; GAN, generative adversarial network; ST-GCN, spatial–temporal graph convolutional network. NR: not reported.

Table S2. PRISMA checklist.

Section and Topic	Item	Checklist item	Location where item is reported
**TITLE**			
Title	1	Identify the report as a systematic review.	Page 1
**ABSTRACT**			
Abstract	2	See the PRISMA 2020 for Abstracts checklist.	Page 7
**INTRODUCTION**			
Rationale	3	Describe the rationale for the review in the context of existing knowledge.	Line 39 - 85
Objectives	4	Provide an explicit statement of the objective(s) or question(s) the review addresses.	Line 86 - 89
**METHODS**			
Eligibility criteria	5	Specify the inclusion and exclusion criteria for the review and how studies were grouped for the syntheses.	Line 102 - 110
Information sources	6	Specify all databases, registers, websites, organisations, reference lists and other sources searched or consulted to identify studies. Specify the date when each source was last searched or consulted.	Line 92 - 94
Search strategy	7	Present the full search strategies for all databases, registers and websites, including any filters and limits used.	Line 95 - 99
Selection process	8	Specify the methods used to decide whether a study met the inclusion criteria of the review, including how many reviewers screened each record and each report retrieved, whether they worked independently, and if applicable, details of automation tools used in the process.	Line 102 - 114
Data collection process	9	Specify the methods used to collect data from reports, including how many reviewers collected data from each report, whether they worked independently, any processes for obtaining or confirming data from study investigators, and if applicable, details of automation tools used in the process.	Line 112 - 114
Data items	10a	List and define all outcomes for which data were sought. Specify whether all results that were compatible with each outcome domain in each study were sought (e.g. for all measures, time points, analyses), and if not, the methods used to decide which results to collect.	Line 122 - 126
	10b	List and define all other variables for which data were sought (e.g. participant and intervention characteristics, funding sources). Describe any assumptions made about any missing or unclear information.	Line 127 - 130
Study risk of bias assessment	11	Specify the methods used to assess risk of bias in the included studies, including details of the tool(s) used, how many reviewers assessed each study and whether they worked independently, and if applicable, details	N/A

		of automation tools used in the process.	
Effect measures	12	Specify for each outcome the effect measure(s) (e.g. risk ratio, mean difference) used in the synthesis or presentation of results.	N/A
Synthesis methods	13a	Describe the processes used to decide which studies were eligible for each synthesis (e.g. tabulating the study intervention characteristics and comparing against the planned groups for each synthesis (item #5)).	Line 122 - 123
	13b	Describe any methods required to prepare the data for presentation or synthesis, such as handling of missing summary statistics, or data conversions.	Line 122 - 123
	13c	Describe any methods used to tabulate or visually display results of individual studies and syntheses.	Line 122 - 123
	13d	Describe any methods used to synthesize results and provide a rationale for the choice(s). If meta-analysis was performed, describe the model(s), method(s) to identify the presence and extent of statistical heterogeneity, and software package(s) used.	N/A
	13e	Describe any methods used to explore possible causes of heterogeneity among study results (e.g. subgroup analysis, meta-regression).	N/A
	13f	Describe any sensitivity analyses conducted to assess robustness of the synthesized results.	N/A
Reporting bias assessment	14	Describe any methods used to assess risk of bias due to missing results in a synthesis (arising from reporting biases).	N/A
Certainty assessment	15	Describe any methods used to assess certainty (or confidence) in the body of evidence for an outcome.	N/A
**RESULTS**			
Study selection	16a	Describe the results of the search and selection process, from the number of records identified in the search to the number of studies included in the review, ideally using a flow diagram.	N/A
	16b	Cite studies that might appear to meet the inclusion criteria, but which were excluded, and explain why they were excluded.	N/A
Study characteristics	17	Cite each included study and present its characteristics.	Line 153 – 287 and Supplementary Materials
Risk of bias in studies	18	Present assessments of risk of bias for each included study.	N/A
Results of individual studies	19	For all outcomes, present, for each study: (a) summary statistics for each group (where appropriate) and (b) an effect estimates and its precision (e.g. confidence/credible interval), ideally using structured tables or plots.	N/A

Results of syntheses	20a	For each synthesis, briefly summarise the characteristics and risk of bias among contributing studies.	N/A
	20b	Present results of all statistical syntheses conducted. If meta-analysis was done, present for each the summary estimate and its precision (e.g. confidence/credible interval) and measures of statistical heterogeneity. If comparing groups, describe the direction of the effect.	N/A
	20c	Present results of all investigations of possible causes of heterogeneity among study results.	Line 404 and 416
	20d	Present results of all sensitivity analyses conducted to assess the robustness of the synthesized results.	N/A
Reporting biases	21	Present assessments of risk of bias due to missing results (arising from reporting biases) for each synthesis assessed.	N/A
Certainty of evidence	22	Present assessments of certainty (or confidence) in the body of evidence for each outcome assessed.	N/A
**DISCUSSION**			
Discussion	23a	Provide a general interpretation of the results in the context of other evidence.	Lin 357 - 388
	23b	Discuss any limitations of the evidence included in the review.	Line 521 - 535
	23c	Discuss any limitations of the review processes used.	Line 440 - 442
	23d	Discuss implications of the results for practice, policy, and future research.	Lin 461 - 403
**OTHER INFORMATION**			
Registration and protocol	24a	Provide registration information for the review, including register name and registration number, or state that the review was not registered.	Line 92
	24b	Indicate where the review protocol can be accessed, or state that a protocol was not prepared.	N/A
	24c	Describe and explain any amendments to information provided at registration or in the protocol.	N/A
Support	25	Describe sources of financial or non-financial support for the review, and the role of the funders or sponsors in the review.	Line 447
Competing interests	26	Declare any competing interests of review authors.	Line 448
Availability of data, code and other materials	27	Report which of the following are publicly available and where they can be found template data collection forms; data extracted from included studies; data used for all analyses; analytic code; any other materials used in the review.	Line 568
